# Longitudinal Change in Rumen-Associated Bacterial Communities Following Aso Limonite Supplementation in Japanese Brown Cattle

**DOI:** 10.3390/ani16091419

**Published:** 2026-05-06

**Authors:** Kentaro Harakawa, Kazuhiko Imakawa

**Affiliations:** Research Institute of Agriculture, Tokai University, Kumamoto 862-8652, Japan; 3mtld001@tokai.ac.jp

**Keywords:** Kumamoto strain of Japanese Brown (JBRK) cattle, Aso limonite, rumen-associated bacterial communities, buccal swab, peripartum period, longitudinal analysis, calf growth, maternal supplementation

## Abstract

Aso limonite, a natural volcanic mineral, has been used as a cattle supplement in the Aso region of Japan; however, its effects have not been well-characterized. This study examined whether Aso limonite supplementation from 105 days prepartum to 90 days postpartum changed rumen-associated bacterial communities and calf growth in the Kumamoto strain of Japanese Brown cattle. Buccal swab-derived bacterial profiles were regarded as proxies of examining rumen-associated bacterial communities. Diversity analysis detected prepartum differences between the control and limonite groups. Primary longitudinal screening further identified rumen-associated, oral-associated and archaea subsets from buccal swab-derived microbiota. Within the rumen-associated subset, *Blastomonas* and *Denitromonas* emerged as representative taxa showing interaction-associated signals at day −45 in the limonite group, whereas *Adlercreutzia* showed different pattern at day −15 in the control group. Additionally, heatmap and network analyses showed that these taxa were found in distinct broader rumen-associated bacterial communities rather than a single common taxon. In parallel, calves born to limonite-fed dams were heavier than those from control dams between days 28 to 84 postpartum. These findings indicate that maternal limonite supplementation was associated with selective changes in rumen-associated bacterial communities together with greater early calf body weight.

## Simple Summary

Aso limonite, a natural volcanic mineral traditionally used in the Aso region of Japan, is believed to improve maternal condition and calf growth. In this study, we tested whether feeding Aso limonite to Japanese Brown cows from late pregnancy to early lactation was associated with changes in rumen-associated bacterial communities and calf weight. Because direct rumen sampling is difficult on commercial farms, buccal swabs were used as a proxy to examine rumen-associated bacterial profiles. The results showed that limonite-related changes were concentrated in the rumen-associated bacterial subset rather than across the whole rumen microbiota. Several representative bacterial taxa showed distinct time-dependent patterns, and heatmap and network analyses indicated that these changes were associated with different bacterial community structures. In addition, calves born to limonite-fed dams were heavier than those from the control group. These findings suggest that maternal limonite supplementation was associated with selective changes in buccal swab-inferred rumen-associated bacterial communities and greater early calf body weight in this small exploratory field study.

## 1. Introduction

The maintenance of animal health to prevent diseases and other physiological risks is required for efficient and sustainable livestock production. Accumulated data show clear symbiotic links between ruminants and their rumen microbiome. These links affect not only nutrient utilization in the dam but also nutrient transfer to the offspring, thereby influencing growth and lifetime performance. Among the microbial communities inhabiting ruminants, the rumen microbiota has been actively investigated for its key role in nutrient utilization and metabolic regulation. In particular, the rumen microbiota digests and ferments ingested feed to produce short-chain fatty acids (SCFAs), which are absorbed through the ruminal epithelium. The composition of the rumen microbiota varies depending on the type, quality, and amount of feed provided. Nevertheless, approximately 80% of core rumen bacterial taxa are consistently present across ruminant species and contribute to host nutrition [1,2]. Some bacterial taxa of ruminants have also been proposed as potential indicators for health status [3,4].

Like other eutherian species, ruminant dams during late gestation exhibit marked changes in immune function and reduced inflammatory responsiveness that support fetal development [5]. In ruminants, these physiological changes may also be influenced by the composition and temporal dynamics of rumen bacterial communities. Previous studies in Holstein cows have also shown that the periparturient period can be accompanied by changes in rumen bacterial structure and/or function, although the magnitude and similarity of these changes vary among studies [4,6,7]. Together, these findings suggest that maternal microbial dynamics during late gestation and/or early lactation may influence neonatal microbial colonization and early-life growth [8,9]. In addition to the nutrients provided by feed, the supplementation of essential minerals is considered important in maintaining maternal health [10,11].

In Japan, rumen microbial studies have been conducted mainly in Japanese Black cattle and have focused on dietary adaptation, microbial ecology, and methane-related traits during the growing and fattening periods [2,12,13]. However, few studies have followed the same breeding cows and their calves longitudinally under commercial-farm conditions. In the Aso region, Aso limonite, a natural mineral derived from the volcanic caldera of Mt. Aso, Japan, that mainly consists of Fe_2_O_3_ and SiO_2_ [14], has long been used empirically as a feed additive with the expectation that it may improve maternal condition after calving and calf health. We previously reported that Aso limonite supplementation at 50 g/day during the prepartum period was associated with altered buccal swab-derived microbial patterns in Japanese Brown cattle [15].

Repeated direct sampling of rumen contents is difficult under commercial-farm conditions, especially during late gestation and early lactation. Buccal or oral swab sampling has been used as a practical non-invasive proxy for rumen microbial profiling in cattle, because it can recover rumen-associated signals, although such samples also contain oral-associated taxa and should not be regarded as direct equivalents of rumen contents [16,17,18,19,20]. Our hypothesis was that limonite supplementation would improve the cattle condition during peripartum periods and calf growth, which can be determined through the examination of the rumen-associated microbiome. In the present study, we extended our previous prepartum observations [15] by continuously monitoring the same dams throughout the postpartum period. Therefore, our objective was to examine whether Aso limonite supplementation was associated with longitudinal changes in buccal swab-inferred rumen-associated bacterial communities during the peripartum period in the Kumamoto strain of Japanese Brown (JBRK) cattle. We also examined whether calf weight differed between the control and limonite groups in this small exploratory field study.

## 2. Materials and Methods

### 2.1. Animals, Management, Experimental Design and Calf Weight Measurement

**Figure 1:**
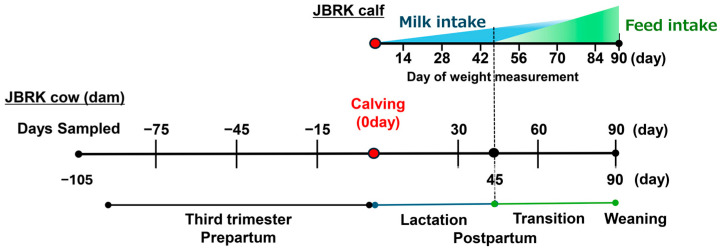
Experimental design and longitudinal sampling scheme. JBRK cows received Aso limonite from 105 days before calving until 90 days postpartum. Calves depend on milk until day 45, from which creep feeder were set thereafter (dashed line). Buccal swab samples were collected from dams at days −75, −45, −15, 30, 60, and 90 relative to calving. Calf body weight was measured from day 14 to day 84. Lactation, transition, and weaning periods are indicated.

Cattle at a commercial farm (Kumamoto Aso Kenmin-farm Co., Ltd., Kumamoto, Japan) housed in a stall barn year-round were used in this study. About two weeks before expected calving, cows were placed in a free barn where cows calved, and cow–calf pairs were kept 10 days to two weeks in this free barn. A cow and calf pair were then placed in a different free barn with other cow–calf pairs until weaning on day 90 (Figure 1). From September 2022, seven pregnant cattle (46.0 ± 22.4 months of age), about 105 days before calving, were enrolled to the study as the control (*n* = 3) and limonite supplemented (*n* = 4) groups and all cows had healthy calves, and thus these cows and calves were continuously monitored 90 days postpartum, for a total of a 195-day period. All dams and calves remained clinically healthy throughout the study, as confirmed by experienced veterinarians.

Buccal swab sampling of dams was performed during the prepartum period (days −75, −45 and −15, day 0 = day of calving) and the postpartum period (days 30, 60 and 90) (Figure 1). Because we sought to minimize stress during the late gestation and early lactation periods, postpartum sampling started on day 30.

Body weights of calves were recorded biweekly from 14 to 84 days postpartum, and values were summarized as mean ± standard deviation (SD).

### 2.2. Feeds and Aso Limonite Supplementation

The cows were fed whole crop silage made from rice, or Italian ryegrass (10–13 kg/day) and concentrate feed (Nippon Nosan Kogyo Co., Ltd., Yokohama, Japan) at 1–2.5 kg/day prepartum and 2.5 kg/day postpartum. Identical amounts were fed twice a day at 08:00 and 17:00. The composition and nutrient information of the roughage and concentrate feed are shown in Table S1. Amounts of feed, which meet beef cattle production, are based upon the Japanese feeding standard for cattle [21]. Nutritional compositions of the roughages were crude protein (CP; 8.5% or 12.7%; rice or Italian ryegrass, respectively), ether extract (EE; 4.1% or 2.6%), acid detergent fiber (ADF; 33.5% or 28.4%), neutral detergent fiber (NDF; 53.2% or 63.4%), crude ash (CA; 13.6% or 13.0%), total digestible nutrients (TDN; 48.8% or 60.6%), digestible energy (DE; 2.15 Mcal/kg or 2.67 Mcal/kg), metabolizable energy (ME; 1.73 Mcal/kg or 2.23 Mcal/kg). Aso limonite (50 g dry powder/day; Japan Limonite Co., Kumamoto, Japan) was mixed into the morning concentrate for only dams throughout the study, consistent with our previous prepartum study [15].

During the active lactation period (0–45 days postpartum), calves were primarily nursed by their dams. From 45 to 90 days postpartum (transition period), frequency of milk intake gradually decreased while calves had free access to concentrate feed supplied through a creep feeder (approximately 300–600 g/head/day) and whole crop silage until weaning on day 90 (Figure 1). The cows and calves had free access to water.

### 2.3. Buccal Swab Collection and DNA Extraction

Buccal swab sampling was done 4–5 h after the morning feeding when highest rumination was observed [18]. Cows were placed in a stanchion, and swabs were collected while their heads were minimally restrained as described by Miura et al. [22] and Harakawa et al. [15]. The samples placed in a 15 mL-tube containing 0.02 M EDTA, 0.02 M sodium citrate trisodium salt and 4.6 M ammonium sulfate and kept in the refrigerated box (4 °C) were carried to the laboratory within 2 h of sampling. DNA from all the samples was extracted and purified in the laboratory as previously described [15].

### 2.4. 16S rRNA Gene Library Preparation and Sequencing

Purified DNA samples were used as templates for amplification of the V3–V4 region of bacterial and archaeal 16S rRNA genes with primers 341F (5′-AATGATACGGCGACCACCGAGATCTACACTCTTTCCCTACACGACGCTCTTCCGATCTCCTACGGGAGGCAGCAG-3′) and 805R (5′-CAAGCAGAAGACGGCATACGAGATNN NNNNGTGACTGGAGTTCAGACGTGTGCTCTTCCGATCT-3′) as described by Takahashi et al. [23]. Barcoded amplicons were sequenced (2 × 301 bp) on an Illumina MiSeq platform (Illumina, San Diego, CA, USA) using the MiSeq Reagent Kit v3 (600 cycles, Illumina). Library preparation and sequencing were performed at a commercial laboratory (Techno Suruga Laboratory Co., Ltd., Shizuoka, Japan).

Data availability: Raw sequence data from the prepartum and postpartum periods have been deposited in the DDBJ Sequence Read Archive under accession DRA018697 and DRA025791, respectively.

### 2.5. Bioinformatics Processing

Using the QIIME2 pipeline, amplicon sequence variants (ASVs) tables were first generated, followed by diversity analyses as whole-community screening. MaAsLin2 in R [24] was then applied as the primary longitudinal screening to identify subset-level, significant and supportive taxa [25]. Significant and supportive taxa identified by this screening step were subsequently evaluated using linear mixed-effects models and Type III ANOVA [26,27]. Finally, heatmap and network analyses were used as exploratory visualizations to summarize broader clustering patterns and local association structures surrounding the significant taxa [28,29]. These network analyses were not intended to infer direct microbial interactions [30,31].

#### 2.5.1. Taxonomic Analysis

Paired-end sequence reads of FASTQ data were merged, denoised, and processed through the QIIME2 pipeline (ver. 2024.5) [32] with the DADA2 plugin [33] to obtain amplicon sequence variants (ASVs). Taxonomic classification was carried out using a pre-trained Naïve Bayes classifier implemented in QIIME2 and trained on the Greengenes database (ver. 13_8) [34]. Taxonomic profiles were summarized at the genus levels (level 6).

#### 2.5.2. Construction of Genus-Level Subsets and Taxonomic Curation

After taxonomic summarization at the genus level, genus-level abundance tables were used to construct subset-specific matrices for downstream analyses. Genus-level taxa were assigned to four analytical categories: all, rumen-associated, oral-associated, and archaea-associated subsets based on taxonomic identity and numerous publications on cattle rumen, buccal, and oral microbiota [16,17,18,19,20], rather than relying only on statistical associations observed in the present dataset. This analytical framework was adopted to distinguish and analyze the rumen-associated microbial subsets/taxa throughout the course of the study. The full genus-level subset assignment table is presented in Table S2.

### 2.6. Statistical Analysis

#### 2.6.1. Diversity Analysis

All samples from the control and limonite groups collected during the prepartum and postpartum periods were included in the analysis, while signals with fewer than 10 reads or zero standard deviation across samples were excluded prior to analysis. Alpha diversity (Shannon, Observed OTUs, and Faith’s PD) was assessed using the Kruskal–Wallis test in QIIME 2 (ver. 2024.5). Beta diversity (Bray–Curtis, Jaccard, weighted UniFrac, and unweighted UniFrac) was evaluated using ANOSIM. Principal coordinate analysis (PCoA) was visualized using Emperor plot in QIIME 2 (ver. 2024.5). In these diversity analyses, a *p*-value of less than 0.05 (*p* < 0.05) was considered statistically significant.

#### 2.6.2. MaAsLin2 and Type III ANOVA

Primary longitudinal screening of genus-level abundance was conducted separately for all-databased (all) and subset-databased (rumen-associated, oral-associated, and archaea-associated) using MaAsLin2 (version 1.18.0) in R (version 4.4.1). Significant taxa identified were further evaluated using linear mixed-effects modeling (LMM).

The response variable was modeled as:

log10 (relative abundance + pseudocount) ~ group × day + (1|animal)(1)

where group, day, and group × day were treated as fixed effects and animal was included as a random intercept to account for repeated sampling. Multiple-testing correction of model-derived *p*-values (*p*) was performed using the Benjamini–Hochberg false discovery rate (FDR) procedure, adjusted values are reported as *q*-value (*q*). Type III ANOVA was used to evaluate fixed effects, and *p*-values (*p*) obtained. Significant taxa were then categorized according to whether they primarily reflected group effects, day effects, or group × day interaction effects.

#### 2.6.3. Heatmaps and Network Analysis

Heatmaps were generated from relative abundance values, which were log-transformed (Z-score) after addition of a pseudocount (1 × 10^−6^), and group–day summary matrices using pheatmap (version 1.0.13). Network objects were generated using igraph (version 2.1.4) and visualized using ggraph (version 2.2.2). Two types of centered association network analyses were performed using Spearman’s rank correlations calculated with Hmisc (version 5.2-3) [35].

#### 2.6.4. Calf Body Weight

Calf body weight was analyzed using a repeated-measures LMM with group, day of age, and their interaction as fixed effects and calf ID as a random intercept. For the main analysis, day of weight measurement was treated as a categorical variable. Estimated marginal means were calculated for each group–day combination, and pairwise comparisons between groups within each day were adjusted by the Holm method. Additionally, mixed models treating day of weight measurement as a continuous variable were fitted to draw supplementary linear trend lines.

## 3. Results

### 3.1. Experimental Animals, Sample Collection, and 16S rRNA Gene Amplicon Sequencing

One cow–calf pair in the limonite group was excluded from the postpartum analysis because the dam did not nurse the calf after delivery and the calf was subsequently reared on milk replacer. As a result, the postpartum comparison included three cow–calf pairs in each group. A total of 11 and 8 buccal swab samples were collected during the prepartum period from the limonite and control groups, respectively, and 9 buccal swab samples per group were collected during the postpartum period. Mean sequence read counts per sample from cow buccal swabs were 24,464 ± 3936 and 29,911 ± 7254 in the prepartum control and limonite groups, respectively, and 19,866 ± 10,918 and 18,298 ± 11,491 in the postpartum control and limonite groups, respectively. Rarefaction analysis indicated sufficient sequencing depth for downstream analyses.

### 3.2. Taxonomic Composition and Diversity Screening Using QIIME2

Regarding the taxonomic composition, a total of 398 taxa were identified, consisting of 389 bacterial taxa and 8 archaeal taxa at the genus level. Taxa with an average relative abundance exceeding 1% during the experimental period were designated as core bacterial taxa. The number of core bacterial taxa in the control group was 19 (81%) in the prepartum period and 19 (74.2%) in the postpartum period. Moreover, 18 taxa (60.5%) of the limonite group were detected in the prepartum period, and 17 taxa (59.9%) were detected in the postpartum period (Figure S1, Table S3).

For alpha diversity, scarcity analysis based on observed OTUs showed that all samples reached a plateau, indicating sufficient sequencing depth. The analysis was performed at a sampling depth of 6000 for the bovine rumen microbiome (Figure S2). The control group showed significantly lower values at 30 and 60 days compared to −45 day, indicating a change in diversity from prepartum to postpartum (*p* = 0.049). In contrast, there were no significant differences in the limonite group during the peripartum period. Between-group comparisons showed differences between the limonite group and the control group at 30 and 60 days (*p* = 0.049; Figure S3).

Beta diversity showed a significant difference between the prepartum limonite treated group and the control group (*p* = 0.001–0.009), particularly in Bray–Curtis distance after FDR correction (*q* = 0.006; Table S4). Biplots of principal coordinate analysis (PCoA) were visualized using Emperor, and the top five taxa were found (Figure S4). Four out of the five taxa were reorganized as oral-related taxa.

### 3.3. Rumen-Associated Subset Identified Using Subset Construction and Longitudinal Screening

**Figure 2:**
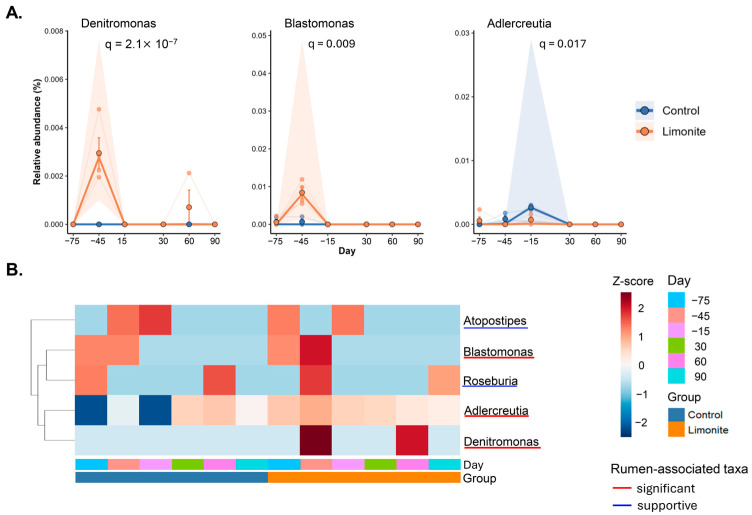
Screening of time-course patterns using MaAsLin2 and visualization with a compact heatmap. (**A**) Time-course patterns of significant taxa identified from the rumen-associated subset. The x-axis indicates sampling day relative to calving, and the y-axis indicates relative abundance (%). Each panel shows the temporal pattern of one significant taxon in the control and limonite groups, with the corresponding *q* value shown in the panel. (**B**) Compact heatmap of rumen-associated taxa across all group–day combinations. Rows significant and supportive rumen-associated taxa, and columns represent group–day combinations. Colors indicate relative Z-score patterns, and the bottom annotation bars indicate day and group. Red underlines indicate significant taxa (*Denitromonas*, *Blastomonas* and *Adlercreutzia*), and blue indicates supportive taxa (*Roseburia* and *Atopostipes*) in the rumen-associated taxa. Only taxa meeting the predefined screening criteria are presented.

Using the MaAsLin2 screening, a subset-specific matrix for downstream analysis identified 385 taxa at the genus levels, which were classified: all (385 taxa), rumen-associated (329 taxa), oral-associated (49 taxa), and archaea-associated (7 taxa). Subset-specific longitudinal screening was conducted across whole database. Within the biologically assigned subsets, the rumen-associated dataset retained 187 tested taxa and yielded three significant rumen-associated taxa (significant taxa) at *q* < 0.05, including two “interaction-associated taxa” of *Blastomonas* (*q* = 0.0023) and *Denitromonas* (*q* = 0.0229) at day −45 in the limonite group, and one “day-associated taxon” of *Adlercreutzia* (*q* = 0.0475) at day −15 in the control group (Figure 2A). In contrast, the oral-associated dataset retained 40 tested taxa and yielded no association (*q* > 0.05). Based on the results from these screenings, the subsequent centered analyses were conducted on the rumen-associated subsets, whereas oral-associated and archaea-associated subsets were treated as supportive analyses.

### 3.4. Rumen-Associated Taxa and Local Association Structure

Following significant taxa were identified as the rumen-associated taxa, the LMM and Type III ANOVA analysis was conducted. These analyses revealed that within-subset group × day effect, *Denitromonas* and *Blastomonas* showed significant signals at day −45 in the limonite group (*q* = 2.10 × 10^−7^ and *q* = 0.009, respectively). In contrast, *Adlercreutzia* showed a different temporal pattern, with a significant signal at day −15 in the control group (*q* = 0.017; Figure 2A, Table S5). Based on these results, *Denitromonas* and *Blastomonas* were regarded as interaction-associated taxa, whereas *Adlercreutzia* was regarded as a day-responsive taxon. Supportive rumen-associated taxa (Supportive taxa) included *Atopostipes* and *Roseburia*, which were identified in the rumen-associated subset (*q* > 0.10; Figure 2B). This heatmap provided a visual summary across all group–day combinations and showed that the significant taxa did not follow a single common temporal pattern.

**Figure 3:**
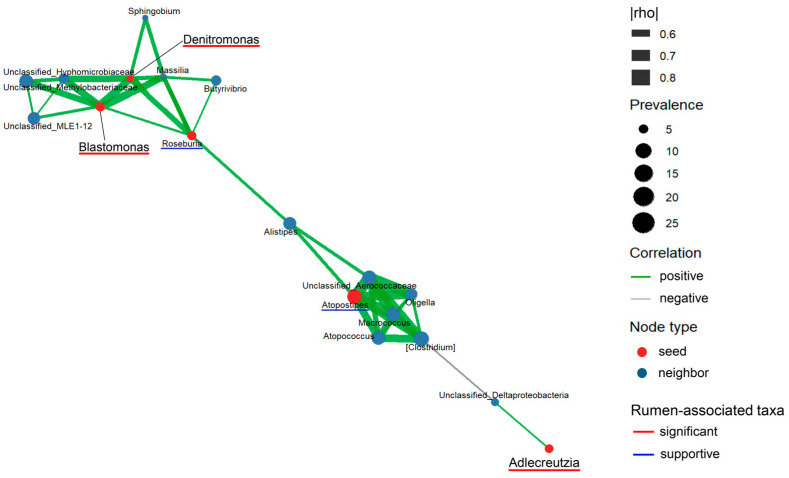
Seed network of significant and supportive rumen-associated taxa with neighboring taxa. The network shows rumen-associated taxa and the taxa directly connected them after correlation filtering. Red nodes indicate seed taxa, and blue nodes indicate neighboring taxa. Node size represents prevalence across group–day combinations. Edge width represents the absolute Spearman correlation coefficient (|rho|), and edge color indicates the sign of the correlation (green, positive; gray, negative). Red underlines mark significant taxa (*Denitromonas*, *Blastomonas* and *Adlercreutzia*), and blue underlines mark supportive taxa (*Roseburia* and *Atopostipes*). Only correlations meeting the predefined filtering criteria are presented.

Local relationships among the rumen-associated taxa were further summarized in the seed network (Figure 3). In this network, *Denitromonas* and *Blastomonas* were positioned within the same local association module, whereas *Adlercreutzia* appeared more peripheral relative to the central cluster. These results indicate that the significant taxa were not only temporally distinct but also differed in their local association structure within the rumen-associated subset.

### 3.5. Expanded Heatmap of the Rumen-Associated and Supportive Taxa

**Figure 4:**
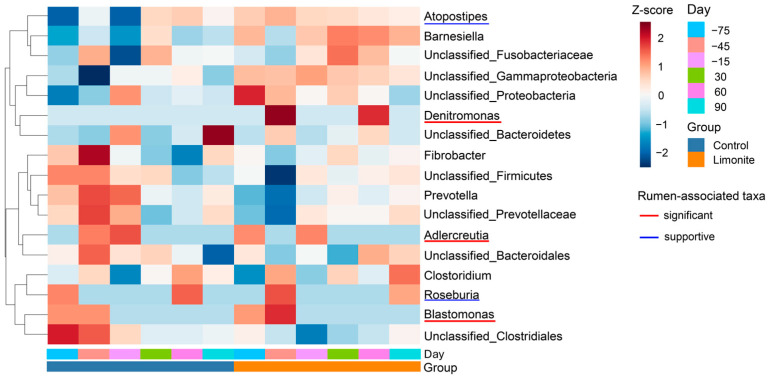
Expanded heatmap of significant and supportive rumen-associated taxa, and broader rumen-associated taxa across group–day combinations. Expanded heatmap showing three significant taxa and two supportive taxa together with 12 broader rumen-associated taxa across all group–day combinations. Rows are arranged by hierarchical clustering to visualize similarities in abundance patterns among significant, supportive and surrounding rumen-associated taxa. Colors indicate relative Z-score patterns across conditions. Red indicates representative significant taxa (*Denitromonas*, *Blastomonas* and *Adlercreutzia*), and blue indicates supportive taxa (*Roseburia* and *Atopostipes*). Only taxa meeting the predefined screening criteria are presented.

The expanded heatmap included three significant taxa, two supportive taxa, and 12 broader rumen-associated taxa across all group–day combinations (Figure 4). The rumen-associated taxa were distributed across different clusters rather than forming a single common response pattern. *Denitromonas* was in an upper cluster containing *Bacteroidetes* and two additional taxa and showed its strongest Z-score at day −45 in the limonite group. In contrast, *Blastomonas* was in a lower cluster together with *Roseburia*, *Clostridium*, and *Clostridiales* and showed its strongest Z-score at day −45 in the limonite group. *Adlercreutzia* was positioned in a different middle cluster associated with *Prevotella*, *Prevotellaceae*, *Fibrobacter*, *Firmicutes*, and *Bacteroidales* under prepartum control conditions, particularly between days −45 to −15. *Atopostipes* were in the upper portion of the heatmap and showed a broader pattern across conditions without a distinct peak. Overall, the Z-score patterns indicated that the rumen-associated taxa presented at least three distinct cluster patterns. Where retained, bootstrap support values supported separation of the major row groupings.

**Figure 5:**
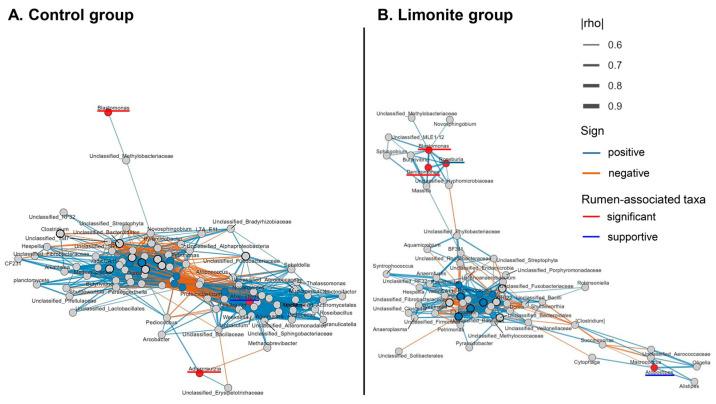
Expanded hub networks in the control and limonite groups. Expanded networks of the rumen-associated subset for the control (**A**) and limonite (**B**) groups. Red nodes indicate significant and supportive taxa, blue nodes indicate hub taxa, and gray nodes indicate other connected taxa. Edge width represents the absolute Spearman correlation coefficient (|rho|), and edge color indicates the sign of the correlation (blue, positive; orange, negative). Only correlations meeting the predefined filtering criteria are retained. The networks show that the focal taxa inhabit different network contexts in the two groups.

These patterns were further examined in the group-specific expanded network as exploratory summaries of centered association patterns (Figure 5A,B). The expanded network contained 121 nodes and 1180 edges in the control group (Figure 5A), whereas the limonite group contained 126 nodes and 473 edges (Figure 5B). The significant and supportive taxa compositions differed between groups: *Adlercreutzia* existed only in the control group (Figure 5A), whereas *Denitromonas* and *Roseburia* were found only in the limonite group (Figure 5B), while *Blastomonas* and *Atopostipes* were present in both groups. Group-specific hub taxa were not significant and supportive taxa in either network and included *Fibrobacter* and *Papillibacter* in both groups, together with additional control-specific hubs such as *Verrucomicrobia* and *Anaeroplasma* (Figure 5A) and limonite-specific hubs such as *YRC22* and *Prevotella* (Figure 5B). Thus, the expanded networks provided additional support that the significant and supportive taxa were associated with different broader structures in the control and limonite groups. For both the seed network (Figure 3) and the expanded networks (Figure 5A,B), only correlations satisfying the predefined filtering criteria were presented, and quantitative summaries of nodes, edges, rumen-associated taxa and hub taxa are provided in Table S6.

### 3.6. Calf’s Body Weight

**Figure 6:**
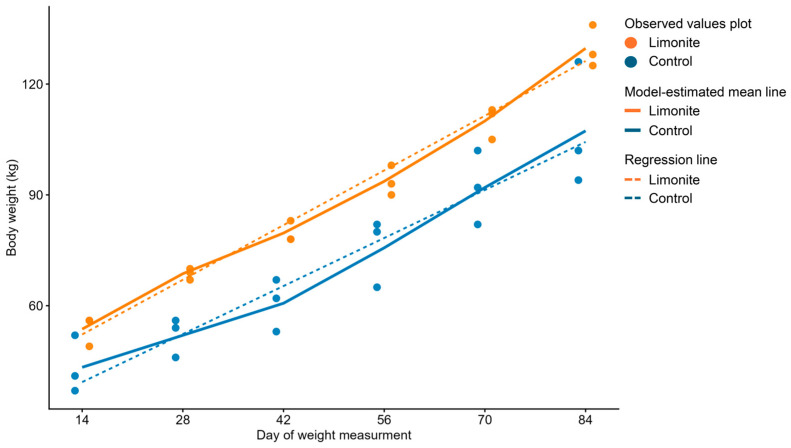
Changes in calf body weight analyzed by repeated-measures linear mixed-effects models. Body weights for individual calves are shown as dots. Solid lines indicate model-estimated marginal means from the repeated-measures linear mixed-effects model treating day of weight measurement as a categorical variable. Dashed lines indicate supplementary linear trend lines from a mixed model treating day of weight measurement as a continuous variable. Each calf was included as a random intercept in both models.

Calves’ body weight measured day 14 to 84 from calving were summarized in Figure 6, Table S7. Repeated-measures linear mixed-effects modeling with day of weight measurement treated as a categorical variable showed significant effects of group and day on body weight, whereas the group × day interaction was not significant (group, *p* = 0.023; day, *p* = 4.12 × 10^−15^; group × day, *p* = 0.531). Thus, body weight increased over time in both groups, and the limonite group showed overall greater body weight than the control group, although the temporal pattern of increase did not differ significantly between groups. Detailed results of the main repeated-measures model are provided in Table S7.

Model-estimated marginal means from the main repeated-measures model were consistently higher in the limonite group than in the control group at all measured time points, increasing from 53.7 kg vs. 43.3 kg average weights on day 14 to 129.6 kg vs. 107.3 kg average weights on day 84 postpartum. Pairwise comparisons between groups within each day are summarized in Table S8. For supplementary visualization of the overall temporal trend, an additional linear mixed-effects model treating day of weight measurement as a continuous variable was also fitted. Detailed results of this supplementary continuous-day model are shown in Table S8.

## 4. Discussion

By examining buccal swab samples, we determined whether Aso limonite supplementation was associated with longitudinal changes in rumen-associated bacterial communities in peripartum JBRK cattle. In alpha diversity, changes were observed in the control group during peripartum periods, but not in the limonite group. Additionally, limonite group’s diversity was lower than control group at 30 days and 60 days postpartum. In beta diversity, microbial communities in the limonite group differed from those of the control group during the prepartum period but not during the postpartum period. The prepartum divergence in these communities could be due to the late stage of gestation, because fetal weight gain accelerates during which rumen microbial structure and function can shift in cattle [4,6,7]. Following alpha and beta diversity analyses, buccal swab associated microbiomes were examined through MaAsLin2 screening, identifying changes in the rumen-associated subset in the limonite group during prepartum period. These results along with those of significant taxa were further evaluated through LMM and Type III ANOVA, finding that the limonite-related effect was not uniformly distributed but was concentrated in the rumen-associated subset. Within this subset, *Blastomonas* and *Denitromonas* emerged as −45 day associated taxa in the limonite group, whereas in the control group, *Adlercreutzia* was found at −15 day. Compact heatmaps and seed network analyses indicated that these changes were not a single common response but rather regarded within different bacterial community structures. Furthermore, calves born to limonite-fed dams were heavier than those in the control group from day 28 to day 84, indicating that maternal limonite supplementation was associated with both subset-specific microbial changes and early growing processes of calves.

These results became more informative when significant taxa were examined within a broader rumen-associated microbiome. The expanded heatmap showed that these taxa, identified as significant taxa, were not grouped into a single common response pattern but were found within different clusters together with distinct neighboring taxa (Figure 4). Rather than forming a single coordinated response block, these significant taxa were distributed across distinct taxonomic neighborhoods, indicating that *Denitromonas, Blastomonas* and *Adlercreutzia* structurally heterogeneous within the broader rumen-associated community. These results were consistent with the group-specific expanded networks, showing changes in the microbial communities with differences in numbers of hubs and nodes (Figure 5). These results suggest that the significant taxa existed within different broader association structures rather than within a single conserved centered organization.

The expanded network analysis supported this interpretation: *Adlercreutzia* was found only in the control group, whereas *Denitromonas* and *Roseburia* existed only in the limonite group, while *Blastomonas* and *Atopostipes* were shared between groups. In addition, the larger number of retained edges in the control group than in the limonite group indicates that the broader association framework surrounding the rumen-associated taxa differed quantitatively as well as qualitatively between groups. These heatmap and network analyses support that significant taxa were selective and embedded within different rumen-associated bacterial organizations rather than forming one common response block. This is consistent with the view that rumen bacterial communities are structured around shared but heterogeneous microbiome [1,2] and with reports that microbial changes during the peripartum period are often taxon-specific rather than uniform [6,7]. These changes observed here may not only be iron-related but may also be in response to the clay mineral-rich nature of limonite, because clay mineral supplementation has been reported to alter rumen-associated microbial communities in cattle [36].

Although numerous examinations have been conducted, buccal swabs are not direct rumen samples but rather contain a mixture of regurgitated rumen-associated materials and oral-associated bacteria. Previous studies in cattle, including work in Holstein cows, have shown that oral or buccal swabs can still recover rumen-associated signals, of which data are not the same as those from directly collected rumen samples in taxonomic composition and analytical resolution [16,17,18,19,20]. In the present study, therefore, this limitation was addressed by applying subset-specific MaAsLin2 screening and LMM and Type III ANOVA evaluation within a conservative subset-based analytical framework. Accordingly, the present findings should be interpreted not as a direct characterization of the whole rumen microbiota, but as evidence that selective rumen-associated bacterial taxa can be detected from buccal swab-derived data when a conservative subset-based analytical framework is applied.

Several limitations should be considered when interpreting these findings. First, the postpartum comparison ultimately involved only three cow–calf pairs per group from a single commercial farm, which limits statistical power and generalizability. The findings should be interpreted as field-based longitudinal evidence obtained under resource-constrained conditions rather than as definitive evidence applicable across broader cattle populations. Second, the study relied on 16S rRNA based profiling of buccal swab-derived samples and did not include direct rumen sampling, functional microbiome analyses, rumen fermentation traits, host metabolic or physiological variables, milk production or milk composition measurements. Moreover, because the present statistical framework was based on relative abundance data rather than a separate CLR-based compositional analysis, the reported microbial associations should be interpreted as exploratory abundance-based patterns. Third, although the expanded heatmap and network analyses were useful for positioning rumen-associated taxa within broader bacterial organization, these visualizations remain exploratory and do not establish causal ecological interactions. Likewise, greater body weight was observed in calves born to limonite-fed dams during the milk-dependent period. However, maternal milk yield or milk composition were not determined in this study, strongly indicating that further experimentation with those measurements is required for definitive conclusion.

Nevertheless, the present study provides field-based longitudinal evidence that together with greater early calf body weight, Aso limonite supplementation was associated with selective changes in buccal swab-inferred rumen-associated bacterial communities in JBRK cattle. Further research with larger number of cattle should be conducted to validate the results from the present investigation, allowing definitive determination on how limonite supplementation improves cattle performances and calf growth through changes in microbial communities as well as milk characteristics.

## 5. Conclusions

Supplementing cows with Aso limonite during late pregnancy to early postpartum period resulted in changes in the rumen-related bacterial community. These changes were concentrated in the rumen-associated bacterial community and were accompanied by early weight gain of calves in this small exploratory field study.

## Data Availability

The data present in this study are deposited to DDBJ Sequence Read Archive under accession DRA018697 and DRA025791 and are available on request from the corresponding author.

## Supplementary Materials

The following supporting information can be downloaded at: https://www.mdpi.com/article/10.3390/ani16091419/s1; Figure S1: Genus-level composition of the buccal swab samples of JBRK cows; Figure S2: Alpha rarefaction curves of the buccal swab samples of JBRK cows based on Observed OTUs; Figure S3: Alpha diversity (Observed OTUs) of the buccal swab samples of JBRK cows with and without Aso limonite supplementation; Figure S4: Principal coordinate analyses (PCoA) of the buccal swab samples of JBRK cows based on three beta diversity (Bray–Curtis) metrics; Table S1: Composition and nutritional content of roughage and concentrate feed given to JBRK cows and calves; Table S2: Genus-level subset assignment for downstream analyses; Table S3: Average relative abundance of bacterial taxa with an average abundance of 1% or more in the buccal swab samples of JBRK cows during the experimental period; Table S4: Pairwise PERMANOVA *p*-values for beta diversity metrics (Bray–Curtis, Jaccard, Unweighted and Weighted UniFrac) across sampling days and treatment groups; Table S5: Statistical summary of significant and supportive taxa were selected from the rumen-associated subset using the MaAsLin2 screening and linear mixed-effects modeling (LMM) and Type III ANOVA support files used to prepare Figure 2; Table S6: Group-specific quantitative summary supporting the expanded hub networks shown in Figure 5; Table S7: Calf body weight changes in calves born to dams with or without Aso limonite supplementation; Table S8: Repeated-measures linear mixed-effects model (LMM) analysis of calf body weight.
