# Supplementary material for: Longitudinal Change in Rumen-Associated Bacterial Communities Following Aso Limonite Supplementation in Japanese Brown Cattle
Source: Animals (Basel). 2026 May 6;16(9):1419. doi: 10.3390/ani16091419 (PMC13162838; doi:10.3390/ani16091419)
Supplement: Supplementary file 1 [file animals-16-01419-s001.zip › animals-4278672-supplementary figures.pdf]

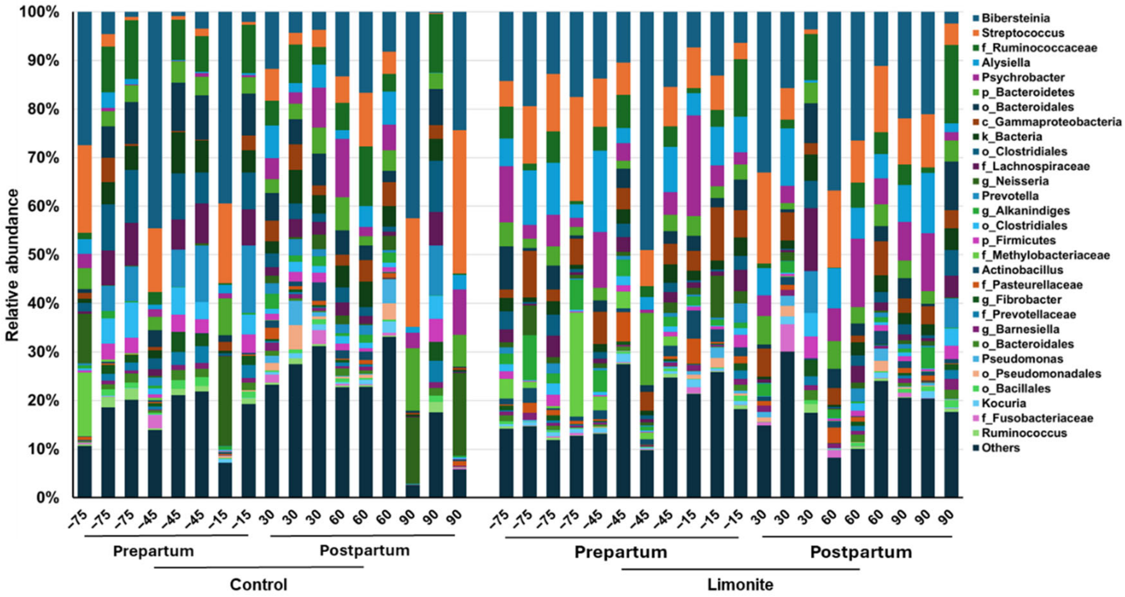

Figure S1. Genus level composition of the buccal swab samples of JBRK cows.
Relative abundance of major taxa (>1% of total reads) in Control and Limonite groups during prepartum and postpartum periods.

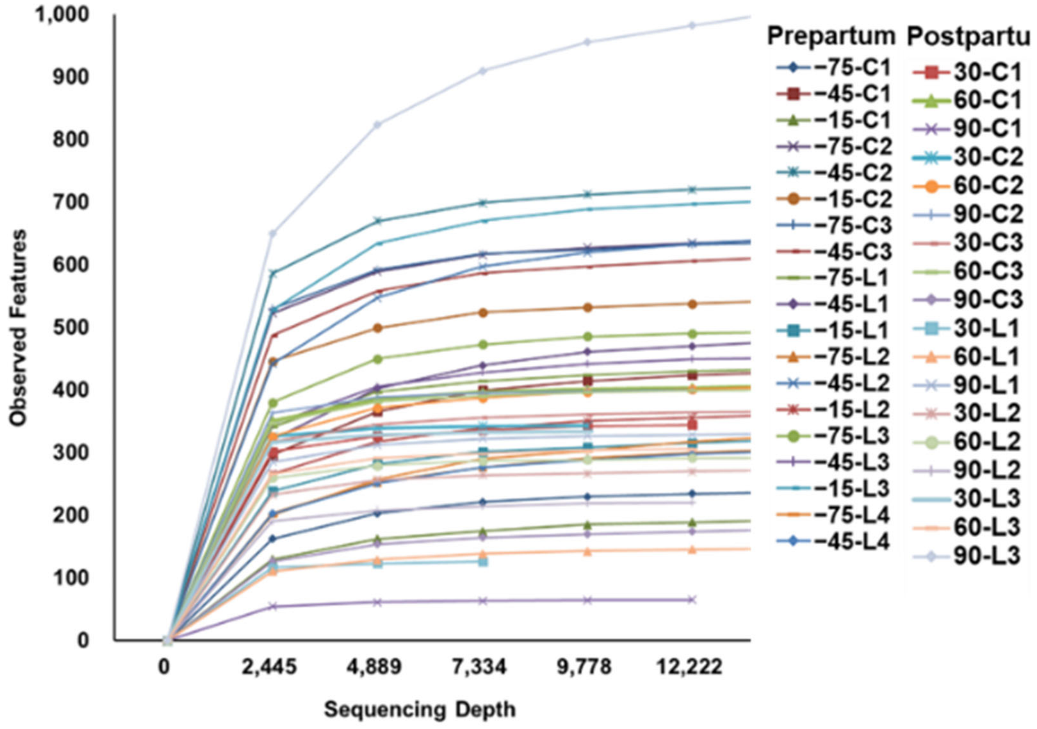

Figure S2. Alpha rarefaction curves of the buccal swab samples of JBRK cows based on Observed OTUs. Buccal swab samples from JBRK cows during all sampling days. “C-” indicates samples from the control group, and “L-” indicates samples from the limonite-supplemented group. Each curve represents an individual sample, with sequencing depth shown on the x-axis. No statistical comparisons were performed using rarefaction curves.

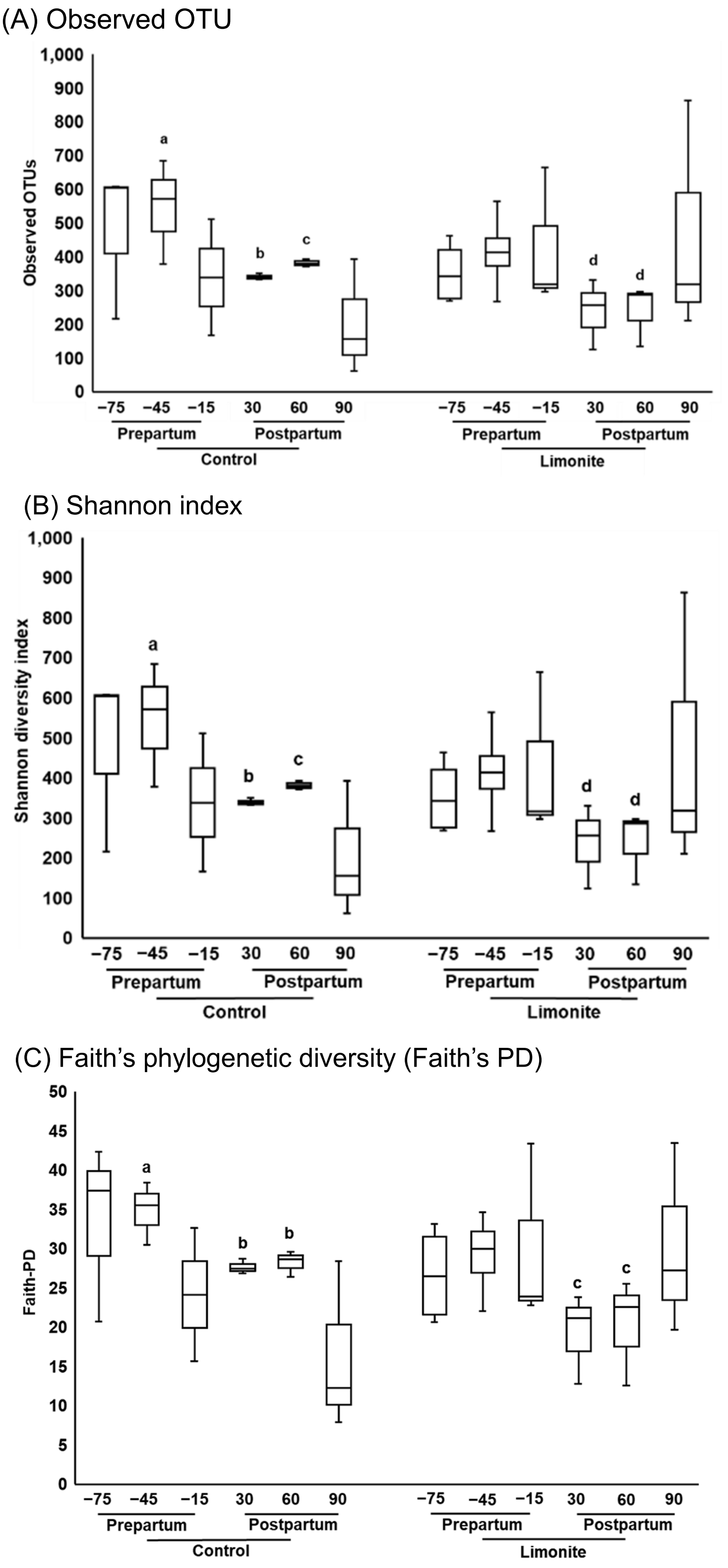

Figure S3. Alpha diversity of the buccal swab samples of JBRK cows with and without Aso limonite supplementation. (A) Observed OTU, (B) Shannon index and (C) Faith's phylogenetic diversity (Faith's PD) during sampling periods. Numbers shown are relative to the parturition. Different letters indicate significant difference ($p < 0.05$, Kruskal–Wallis test). Different letters indicate significant pairwise difference based on Dunn's post-hoc test following the Kruskal–Wallis test ($q < 0.05$, FDR-adjusted). Values represent medians with interquartile ranges.

(A) Prepartum period

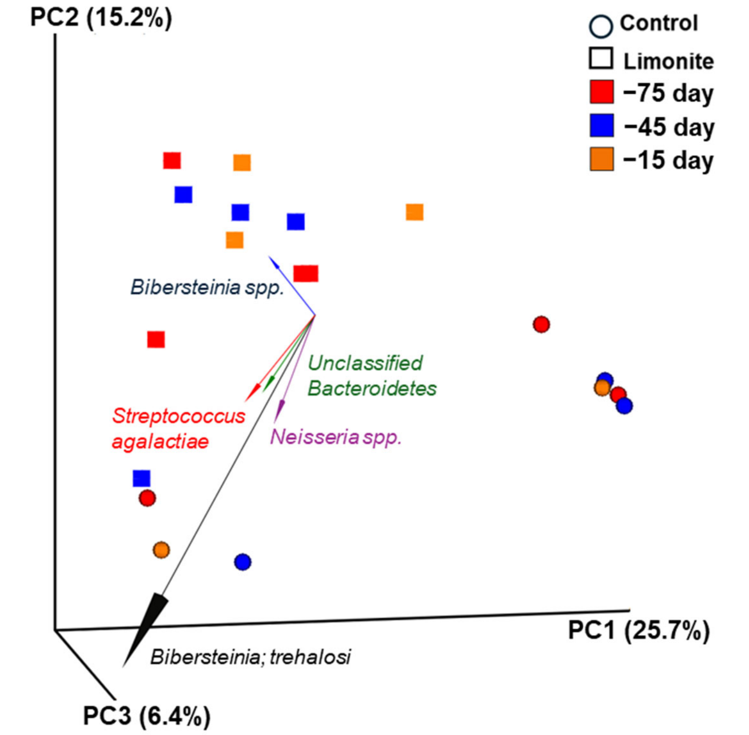

(B) Postpartum period

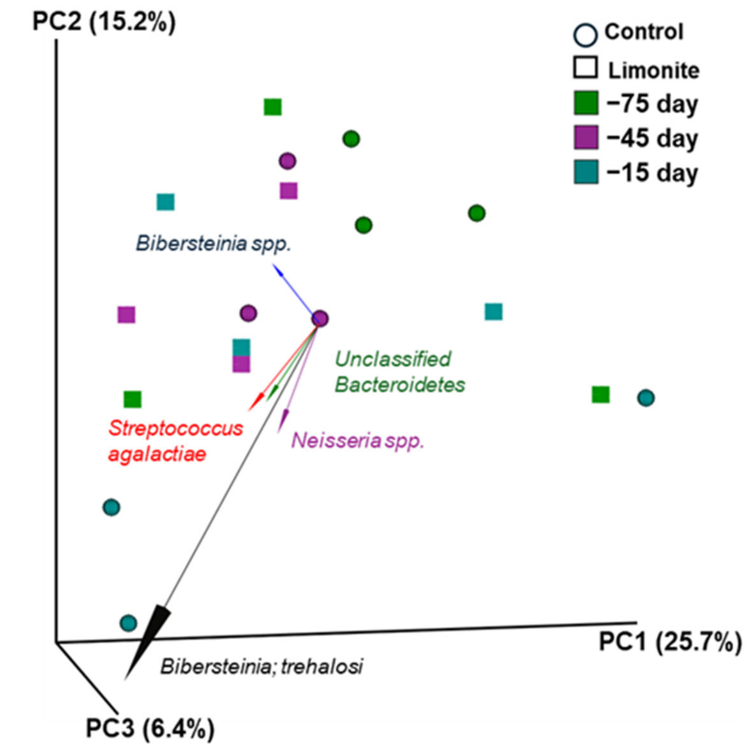

Figure S4. Principal coordinate analyses (PCoA) of the buccal swab samples based on three beta diversity(Bray-curtis) metrics. (A) Prepartum and (B) Postpartum period. Each point represents an individual sample, colored by sampling day and shaped by ○: Control group, □: Limonite group). Arrows indicate genera with the greatest contributions to ordination separation (biplot scores).
